# A Novel Solid Phase Extraction Sample Preparation Method for Lipidomic Analysis of Human Plasma Using Liquid Chromatography/Mass Spectrometry

**DOI:** 10.3390/metabo11050294

**Published:** 2021-05-04

**Authors:** Alex Apffel, Limian Zhao, Mark J. Sartain

**Affiliations:** 1 Agilent Technologies, Santa Clara, CA 95051, USA; mark_sartain@agilent.com; 2 Agilent Technologies, Wilmington, DE 19808, USA; limian_zhao8@agilent.com

**Keywords:** lipidomics, lipids, sample preparation, SPE, mass spectrometry, liquid chromatography

## Abstract

Lipidomic approaches are widely used to investigate the relationship between lipids, human health, and disease. Conventional sample preparation techniques for the extraction of lipids from biological matrices like human plasma are based on liquid-liquid extraction (LLE). However, these methods are labor-intensive, time-consuming, and can show poor reproducibility and selectivity on lipid extraction. A novel, solid-phase extraction (SPE) approach was demonstrated to extract lipids from human plasma using a lipid extraction SPE in both cartridge and 96-well-plate formats, followed by analysis using a combination of targeted and untargeted liquid chromatography/mass spectrometry. The Lipid Extraction SPE method was compared to traditional LLE methods for lipid class recovery, lipidome coverage, and reproducibility. The novel SPE method used a simplified protocol with significant time and labor savings and provided equivalent or better qualitative and quantitative results than traditional LLE methods with respect to several critical performance metrics; recovery, reproducibility, and lipidome coverage.

## 1. Introduction

Lipids comprise a distinct class of small metabolic biomolecules with a diverse range of functions, including storage of energy [1,2], serving as major structural components of biological membranes [3,4,5], and participating in cellular signaling [6,7,8]. Lipidomics is a branch of analytical biochemistry that deals with the large-scale study of cellular lipids in biological systems. Recent advances in analytical technology, including advances in liquid chromatography, mass spectrometry, and informatics, have improved the understanding of critical roles that lipids play in biological systems [9,10]. New analytical capabilities, in turn, have led to increased demand for high throughput and improved reproducibility and sensitivity for lipidomics workflows to allow for larger cohorts in biological studies [11,12,13]. 

One of the primary bottlenecks in any lipidomic workflow is sample preparation. The challenge of a sample preparation technique is to isolate entire classes of lipids while minimizing perturbation to their quantitative representation in the biological sample under study and simultaneously removing matrix co-extractives that interfere with analysis. Traditionally, sample preparation for lipidomic analyses is based on liquid-liquid extraction (LLE) using various solvents or solvent mixtures. Various extraction methods using different organic solvents have been developed and compared in the past [14,15]. The basis of the LLE procedure is to partition lipids into a non-polar organic phase while polar metabolites remain in the polar aqueous phase. Simultaneously, proteins are precipitated, effectively quenching any subsequent metabolic activity. The earliest LLE approach for lipids, still widely used, is the Bligh-Dyer method [14,16,17], which uses chloroform/methanol as the extraction solvent. The Folch method [14,17,18] modified the Bligh-Dyer method in proportions of chloroform/methanol and water used in the extraction. More recently, the Matyash method [14,15,19] and BUME method [20,21,22] substituted the chloroform/methanol (MeOH) with methyl tert butyl ether (MTBE)/MeOH and butanol (BuOH)/MeOH for the sample extraction, respectively. Solvent mixture of MTBE/MeOH is less dense than the aqueous layer, forming a lighter lipid-containing upper layer that is easily separated, whereas chloroform/methanol forms a denser lipid-containing lower layer, which is more difficult to isolate. The BUME method has been further adapted [23,24,25] to a single-phase method using the mixture of BuOH/MeOH, eliminating the need for phase separation. While these methods, particularly the two-phase LLE methods, are well-established and widely used, they are time-consuming and labor-intensive, difficult to automate, and show poor reproducibility in many cases. The single-phase method offers a simplification in usability and adaptability to automation, but it still requires extensive centrifugation to pellet the protein precipitate and may not remove polar interferences efficiently.

There have been reported methods for extracting lipids directly from biological matrices based on solid-phase extraction (SPE) as an alternative to LLE approaches. However, these techniques have either utilized non-specific reversed-phase sorbents [26] or are limited by selectivity, for example, preferentially targeting phospholipids [27,28]. 

In this work, we report a novel solid-phase extraction method using Lipid Extraction SPE cartridges or plates for the quick extraction, purification, and enrichment of a broad range of lipid classes from human plasma. Lipid Extraction SPE cartridges and plates use Enhance Matrix Removal -Lipid (EMR-Lipid) sorbent, which demonstrates selective and efficient interaction with lipid molecules. EMR-Lipid sorbent was first introduced in 2015 [29] as a dispersive SPE (dSPE) sorbent. In 2017, EMR-Lipid cartridges and plates were introduced with a modified sorbent packed in cartridges and plates format [30]. The EMR-Lipid sorbent interaction with lipid molecules is based on the combined mechanism of size exclusion and hydrophobic interaction. Long unbranched hydrocarbon chains on lipid molecules are selectively retained into pores of the EMR-Lipid sorbent, while the other compounds lacking linear acyl chains stay in solution. Currently, the applications of EMR-Lipid sorbent have been primarily focused on “a lipid catch and removal” function, in which lipids from the matrix are retained on the EMR-Lipid cartridge or dSPE sorbent, while the target analytes remain in the sample for further treatment or analysis. 

With Lipid Extraction SPE cartridges, using the EMR-Lipid sorbent for lipid analysis sample preparation introduces a new application area, where lipids become the analytes of interest to be extracted and isolated from other sample matrix interferences. Like traditional SPE, the sorbent’s “catch and release” function is used in these applications. Lipid compounds are retained on the EMR-Lipid sorbent first and then released with the elution solvent. 

This study investigated the use of Lipid Extraction SPE cartridges and plates for lipidomic analysis in human plasma samples by LC-MS and included a comparison to four traditional LLE methods. From the perspective of experimental design, this investigation was conducted in two separate phases. In phase I, an LC-MS/MS method using an LC/TQ (liquid chromatography/tandem quadrupole) instrument with a dynamic Multiple Reaction Monitoring (dMRM) method was used to study recovery and reproducibility. In phase II, a combination of LC-HRMS (High Resolution Mass Spectrometry) or LC-HRMS/MS methods instrument was used for untargeted lipid profiling in human plasma. The four major LLE methods and SPE methods on cartridge and plate were compared in both the recovery and reproducibility test and the untargeted lipid profiling test.

## 2. Results and Discussion

This research aimed to develop and evaluate the novel Lipid Extraction SPE method for preparing human plasma for lipidomics analysis. The results were evaluated by benchmarking against four conventional LLE methods. The strategy was to prepare lipid samples from a standardized plasma sample (SRM 1950) using each method and then compare the lipid profiles from each preparation method with respect to several critical performance metrics: recovery, reproducibility, and lipidome coverage. To focus the comparison on standardized methodologies, widely accepted standard protocols for LLE approaches [14,15,16,17,18,19,20,21,22,23,24,25] and well-characterized reference plasma samples with a sample volume of 100 μL were used.

### 2.1. The SPE Lipid Extraction Method Development

Adapting materials, previously used for removing lipids as unwanted matrix from samples, to a new use for extracting lipids as the primary analytes of interest required method development and optimization. The Lipid Extraction SPE procedure includes three parts: sample loading for lipids retention on sorbent; washing of unwanted matrix components; elution of trapped lipids for recovery.

**Figure 1:**
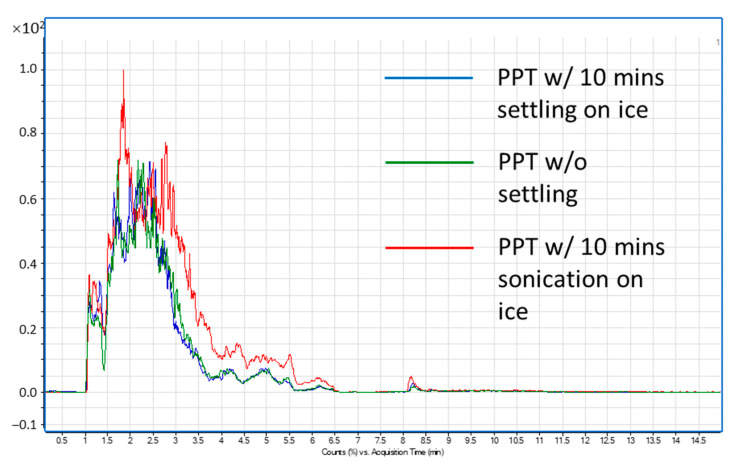
PC/SM profile comparison to demonstrate that sonication on ice after PPT extraction improves lipids extraction efficiency. Blue profile: plasma sample with 10 min of sample mixture sitting on ice for 10 min after PPT extraction. Green profile: plasma sample without settling after PPT extraction. Red profile: plasma sample with 10 min sonication on ice after PPT extraction.

To prepare plasma samples for loading, organic solvent was used to remove proteins and extract most lipids and many matrix co-extractives. Although the traditional methods for lipid extraction typically involve a hydrophobic solvent such as chloroform, the EMR-Lipid sorbent requires a water-miscible solvent for extraction to assure the homogeneity of the mixture with water. Therefore, protein precipitation (PPT) extraction using ACN was used for lipid extraction and protein removal. Efficient protein precipitation is essential to achieve efficient lipid retention on the SPE sorbent since macromolecules like proteins could block EMR-Lipid sorbent pores and subsequently compromise lipid retention. With the efficient protein precipitation, the precipitates were efficiently removed through filtration during the SPE loading step, assuring no large protein molecules getting into sorbent to impact the EMR-Lipid retention for lipid molecules. A high ratio of crashing solvent (95:5 *v*/*v* ACN:MeOH)/plasma (9:1) was used to improve the extraction efficiency for lipid compounds, and cold precipitation solvent was used to improve the complete protein precipitation. MeOH (5%) was added in ACN for precipitation to prevent the generation of large protein precipitate coagulates, which could trap lipids and clog the pipette tip during homogenate transfer. After the addition of precipitation solvent, samples were vortexed for 2 min and sonicated on ice for 10 min. Sonication on ice was to improve the lipid extraction, especially for the lipids potentially trapped within the precipitates. Figure 1 shows the total phospholipid profile comparison for the plasma sample SPE extract using 10-min settling on ice, without settling, and with 10-min sonication on ice. To the fast evaluation and comparison on the different parameter settings’ impact on the lipids’ extraction efficiency, the polar lipid (PL) profile was assessed with LC-MS/MS by monitoring phosphatidylcholine (PC) and sphingomyelin (SM), with a precursor ion scan of *m*/*z* 184.1 The results clearly demonstrate that the improved phospholipids extraction efficiency was achieved when using the 10-min sonication on ice. Cold sonication was used to prevent the potential lipid degradation during sonication. 

It is also crucial to transfer the entire sample homogenate to the SPE cartridge or plate, as some lipids, especially lipoproteins, can be trapped by protein precipitates. Even though the sonication on ice after PPT extraction was helpful to release the trapped lipids, the entire homogenate transfer can further prevent potential lipid loss. As long as the complete sample homogenate is transferred to SPE cartridge or plate, the unreleased trapped lipids can be recovered during the elution step. 

**Figure 2:**
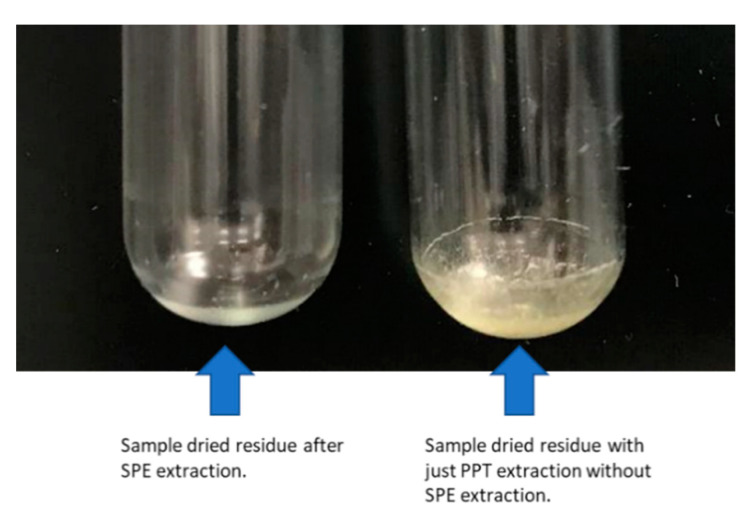
Plasma (100 µL) extract dried residue with SPE extraction (**left**) and with PPT extraction only (**right**).

The loading step allows unwanted matrix co-extractives to pass through the SPE cartridge, including salts and other co-extractives lacking linear acyl chain on the molecular structure. Because the sorbent selectively binds lipids, a washing reagent containing high organic (up to 90%) was used, which allowed more efficient washing to remove other matrix co-extractives. Figure 2 shows a visual comparison of the 100 µL plasma extract dried residue using SPE extraction and PPT extraction only. For the sample treated by SPE procedure, an oily layer of light white was left after the sample being dried. In contrast, for a sample without SPE procedure, much more yellow salts residue was left after drying, indicating significantly more matrix co-extractives in the sample. 

Table 1 shows the results for the elution condition development study. For the preliminary elution investigation, 12 different elution solvents were tested at n = 2 for rapid evaluation. The preliminary study indicated that the efficient lipid elution required a mixture of methanol and a hydrophobic solvent. The further elution investigation included the different ratio for the most promising mixtures, chloroform/MeOH, MTBE/MeOH, DCM/MeOH and 1-chlorobutane/MeOH (shown in Table 1). The two solvent mixtures 1:1 chloroform/methanol and 1:2 DCM/MeOH provided high recoveries (>110%) and reproducibility (RSD < 1%), indicating the best extraction efficiency. Over 100% recovery on PLs also demonstrates the recovery of trapped lipids from protein precipitates during sample elution. This verifies the importance of the complete homogenate transferring after PPT extraction. The 1:2 DCM/MeOH was chosen due to its lower toxicity compared to 1:1 chloroform/MeOH mixture. To ensure maximized recovery, a second aliquot of elution solvent was used.

### 2.2. Comparison of Lipid Extraction Methods

#### 2.2.1. Lipid Class Recovery and Reproducibility

**Figure 3:**
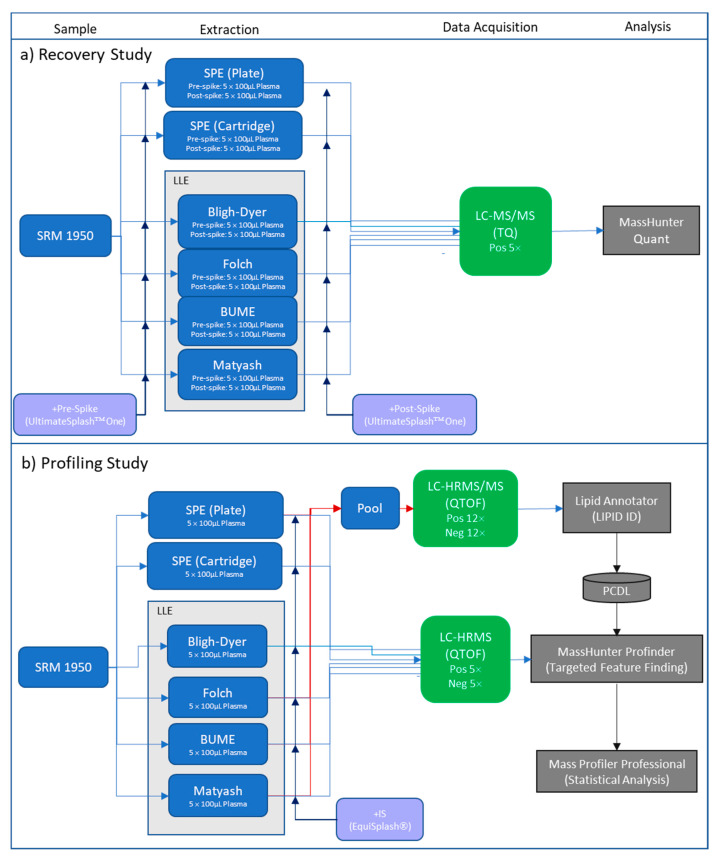
Experimental design. (**a**) recovery study (**b**) profiling study.

As described in the introduction, this method evaluation study was conducted with two approaches: quantitative lipid recovery evaluation of the representative deuterium-labeled lipid internal standards using a targeted LC-MS/MS method on the LC/TQ instrument and qualitative lipid profiling using LC-HRMS and LC-HRMS/MS on the LC/QTOF instrument. The overall experimental design of the two phases is shown in Figure 3.

**Figure 4:**
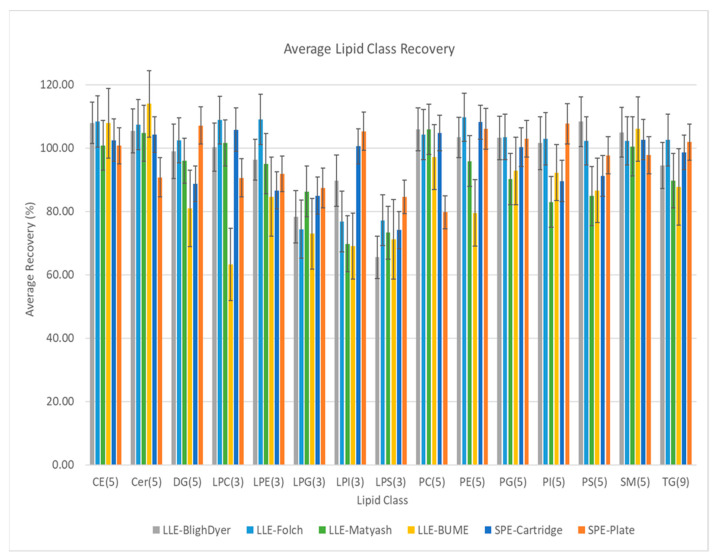
Average recovery of deuterium-labeled lipid internal standards by lipid class based on a targeted LC/TQ workflow. Each bar represents the average of (n) lipids × 5 preparation replicates × 5 MS replicates. Error bars are calculated from average RSD on abundance measurements in ratio. Cholesterol esters (CE), ceramides (Cer), diacylglycerides (DG), lysophosphocholine (LPC), lysophosphoethanolamine (LPE), lysophosphoglycerol (LPG), lysophosphoinosotol (LPI), lysophosphoserine (LPS), phosphatidylcholine (PC), phosphatidylethanolamine (PE), phosphatidylgycerol (PG), phosphatidylinositol (PI), phosphatidylserine (PS), sphingolmyelin (SM), triacylglycero l(TG). Number of individual lipids in each class are shown parenthetically.

In the phase I study, the recovery of 63 deuterium-labeled lipid compounds, representing lipids from 15 classes, were compared for each preparation method using LC-MS/MS dMRM. Figure 4 summarizes these data for 63 lipid compounds averaged across all the lipids within each of the 15 represented lipid classes.

The recovery data for the individual lipids in each class are shown in a box and whisker plot format in Supplementary Figure S1a,b. The results demonstrate that for all classes, the SPE extraction protocols, on both cartridges and plates, yield greater than 70% recovery. Compared to the LLE methodologies, the Lipid Extraction SPE methods yield equivalent or better results within the error of the measurements. Several of the lysophospholipid classes, including lysophosphoglycine (LPG), lysophosphoinositol (LPI) and lysophosphoserine (LPS), show relatively lower recoveries (less than 80% recovery) using LLE methods. 

Compared to the recoveries between those from SPE cartridges and SPE plate methods, the average recoveries show good consistency for all lipids classes, except for PCs with over 20% difference on average recoveries of five PC compounds. One of possible reasons for this deviation could be related to the slight sorbent bed mass between 1 mL cartridge (40 mg) and 96-well plate (60 mg). The other possible reason could be the different samples size on protein precipitation extraction. For 1 mL SPE protocol, the 100 µL of plasma was extracted by 900 µL of cold solvent individually. For 96-well plate protocol, the 1 mL of plasma was extracted by 9 mL of cold solvent in bulk. After sample extraction, 1 mL of homogenate was transferred to each well. The PPT extraction in bulk was explicitly used for this experiment to exclude the deviations during PPT extraction for the comparison. However, such bulk protein precipitation may have resulted in differences in protein precipitation efficiency and, consequently, the following potential lipid retention. The bulk PPT extraction, however, is not recommended for standard sample prep on the plate. Samples should be extracted individually for each well. Even with over 20% of differences in the average results for PC compounds, the recoveries from both SPE cartridges and plates were over 80%, considered acceptable. 

The error bars shown in Figure 4 represent %RSD of the individual area measurements that were used to calculate the average recovery of individual lipid compounds belonging to each class. The average %RSD of the LC-MS/MS analytical method based on replicate injections same sample from all extraction methods was 2.8%. The %RSD for the recoveries based on the five replicate sample preparations using the Lipid Extraction SPE cartridge and plate methods were both 5.9%, averaged across all 63 lipids. In comparison, the four conventional LLE methods, Bligh-Dyer, Folch, Matyash, and BUME methods, yielded total average %RSDs of 7.3%, 7.9%, 8.3%, and 10.8%, respectively, each for five replicate sample preparations. The SPE methods demonstrated equivalent or improved reproducibility compared to conventional LLE methods. The improved reproducibility is attributed to the simplified procedure and easy operations with the elimination of phase separation and manual transfer steps, which reduces human errors and system deviations common with manual sample preparation.

#### 2.2.2. Lipid Class Profiling

**Figure 5:**
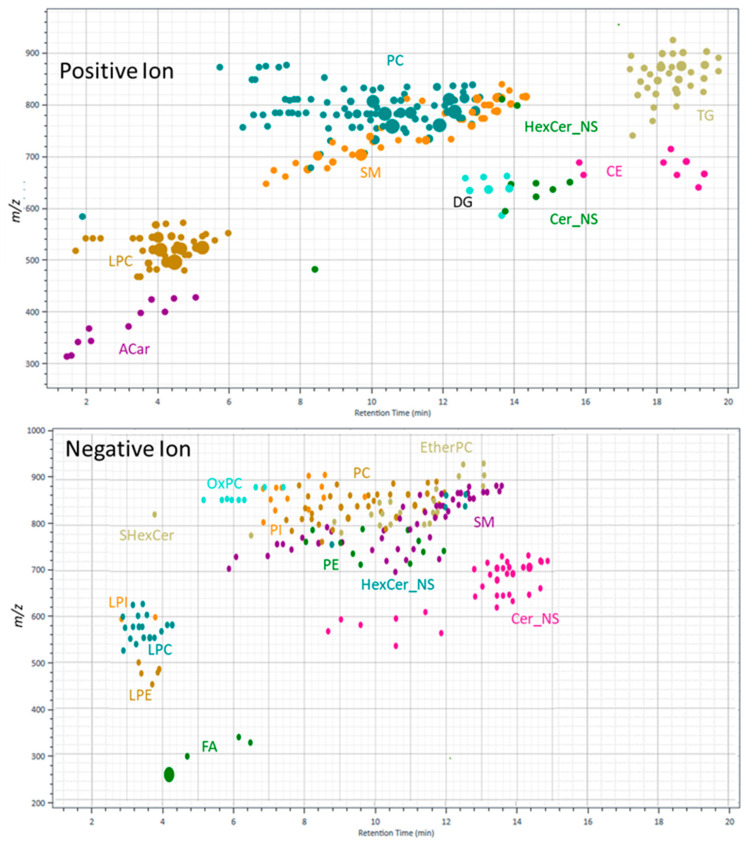
Retention time (RT) vs. mass (*m*/*z*) for LC-HRMS/MS based on Lipid Annotator software annotations. Data generated from 12 iterative HRMS/MS acquisitions on pooled plasma extract samples in both positive and negative ion modes. Bubble size represents log-scaled relative signal abundance. Acylcarnitine (Acar), ceramide non-hydroxyfatty acid-sphingosine (Cer_NS), diglyc-eride (DG), ether phosphatidylcholine (EtherPC), fatty acid (FA), hexosyl-ceramide (HexCer_NS), lysophosphatidylcholine (LPC), lysophosphatidylethanolamine (LPE), lysophosphatidylinisitol (LPI), oxidized phosphatidylcholine (OxPC), phosphatidylcholine (PC), phosphatidylethanolamne (PE), phosphatidylinositol (PI), sulfatides (Shex_Cer), sphingomyelin (SM), triglyceride (TG).

The untargeted LC-HRMS and LC-HRMS/MS approaches on LC/QTOF were used to evaluate lipid coverage. The first step in the analytical workflow was to establish an accurate mass and retention time database from LC-HRMS/MS data using a pooled sample comprised of aliquots from each preparation method. The results of a pooled sample defined all potential lipids for subsequent profiling are shown in Figure 5. Based on 12 iterative injections in each polarity, 243 lipid compounds from 10 classes were annotated in positive mode, and 206 lipid compounds from 13 classes were annotated in negative mode. These data were used to create a database of accurate mass and retention times that served as the basis for annotating subsequently acquired LC-HRMS data. 

**Figure 6:**
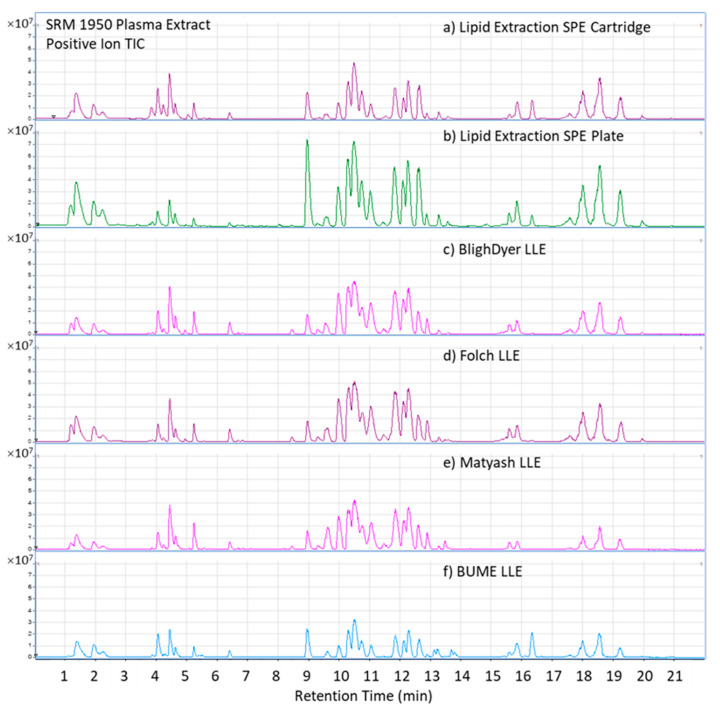
LC-HRMS positive ion total ion chromatograms (TIC) chromatograms for lipid extracts of Scheme 1950. Plasma. (**a**) Lipid Extraction SPE (solid phase extraction) cartridge, (**b**) Lipid Extraction SPE plate, (**c**) BlighDyer LLE (liquid-liquid extraction), (**d**) Folch LLE, (**e**) Matyash LLE, (**f**) BUME LLE.

Representative positive mode total ion chromatograms (TICs) for the human plasma extract prepared by different methods are shown in Figure 6. It is clear from the TIC comparison that the general pattern and abundances of the lipids extracted by each method are very similar, with some visually noticeable differences. For example, for all methods, the TG profiles within retention time (RT) window 17–20 min appear to have a very similar pattern with variations on the peaks intensity by various methods. However, the TIC chromatograms in RT window 15–17 min, comprising predominantly cholesterol esters, show similarities between BUME and SPE methods and between Bligh-Dyer, Folch and Matyash LLE methods. In contrast, the TIC chromatograms in the RT window 8–14 min, where primarily PCs and SMs are eluted, show observable variations across all methods.

**Figure 7:**
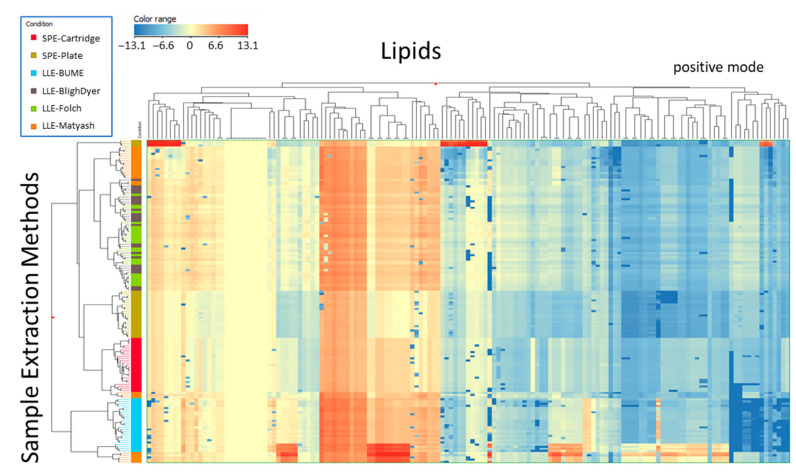
Unsupervised hierarchical lipid clustering by different sample preparation methods for positive ion mode LC-HRMS data. Each extraction method is represented by 5 preparation replicates × 5 analytical replicates. Log2 transformed abundance were normalized with respect to deuterium-labeled lipid class standards (EquiSplash^®^).

These similarities are illustrated more globally by the unsupervised hierarchical clustering of each of the samples on both the sample preparation methods and the individual annotated lipids (Figure 7). Note that in this diagram, each sample preparation method is represented by five preparation replicates × five analytical replicates. In positive mode, the features from the two Lipid Extraction SPE methods generally cluster very closely together. Similarly, the conventional LLE methods generally cluster together. Similar patterns are seen from the negative mode LC-HRMS data shown in Supplementary Figure S2.

**Figure 8:**
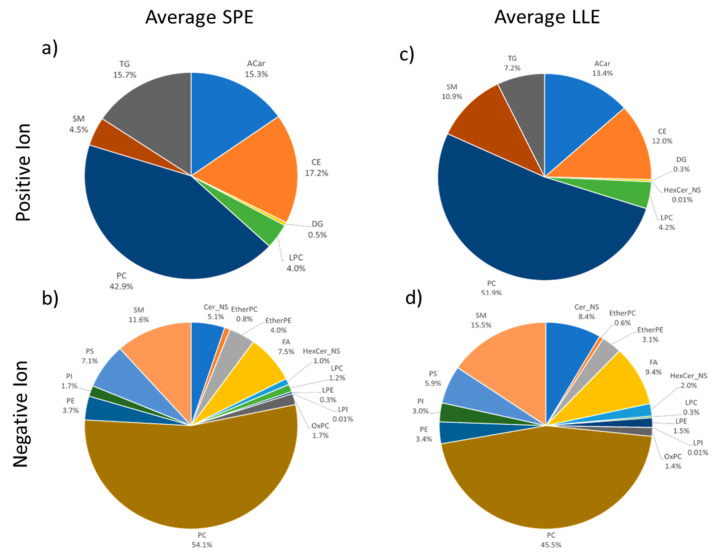
Average lipid class distribution based on calibrated molar concentration (nmol/L) from LC-HRMS profiling study. Average SPE data represent the average of both SPE cartridge and SPE plate × five preparation replicates × five MS replicates. Average LLE data represent the average of four LLE methods × five preparation replicates × five MS replicates. (**a**) Average SPE, Positive Ion, (**b**) Average SPE, Negative Ion, (**c**) Average LLE, Positive Ion, (**d**) Average LLE, Negative Ion.

The comparison of the distribution of different lipid classes between SPE and LLE methods is shown in Figure 8. This comparison is based on the average of the two Lipid Extraction SPE methods and the average of four LLE methods for concentrations calibrated against deuterium-labeled class standards.

For each chart, the concentrations were summed across lipid classes and averaged across five LC-HRMS injection replicates for each of the five preparation replicates. The results for the average SPE in positive (Figure 8a) and negative (Figure 8b) modes were averaged across both SPE methods. The results for the average LLE in positive (Figure 8c) and negative (Figure 8d) modes were averaged across the four LLE methods. The results show high similarity based on the proportions and concentrations of the identified lipid compound classes. The identified lipid compound class distributions are comparable to the consensus distribution for SRM 1950, published by Bowden et al. [31] (data not shown). 

The data shown graphically for the averages of SPE and LLE methods in Figure 8 are broken out for individual methods in Table 2, which lists the number of identified individual lipid compounds and the summed concentrations within each class the positive and negative modes. The total number of individual lipids in each class is less than the maximum number of peaks detectable in the accurate retention time and mass database, due to the stringency filter applied to the analysis.

#### 2.2.3. Comparison Based on Quantitative Method Performance

In this study, Lipid Extraction SPE methods on cartridges and 96-well plates have been demonstrated to offer as the viable alternatives to conventional LLE procedures for lipidomic sample preparation for human plasma, providing comparable or improved method performance. The comparison of various methods was made from several perspectives. On the one hand, quantitative performance metrics such as recovery, reproducibility, and representation of lipid class distributions were made. On the other hand, less tangible, qualitative measures were used to assess the usability of these methods. These include ease of use and throughput. In this section and the next, insights and conclusions from phase I and phase II of the study are integrated and consolidated.

Previous studies have shown variations in the relative representation of specific lipid classes between different sample preparation methodologies [14,15,17,19]. Comparing the recovery of the well-defined set of 63 deuterium labeled lipids in the UltimateSplash™ One internal standard as shown in Figure 4 and Supplementary Figure S1, the difference between the recovery for the samples prepared by the Lipid Extraction SPE method versus LLE methods is comparable to the differences in the recovery across the individual LLE methods. This is also true of other individual lipids within each class, as shown in Supplementary Figure S1a–l. 

Most lipid classes are similarly represented in both their identified lipid compounds numbers and calibrated concentrations, as shown in Figure 7 and Figure 8. There are, however, several notable exceptions. Compared to all LLE methods, the Lipid Extraction SPE prepared samples show a relatively lower abundance of free fatty acids (FAs), ceramides (Cer) and phosphatidylserines (PSs). The reasons for these differences still need further investigation, although it should be noted that only one or two individual lipids represent several classes. The most commonly used approach to lipid quantitation is based on relative quantitation using multiple lipid class-specific isotopic internal standards [15,16]. In the absence of a complete set of isotope-labeled internal standards covering all represented classes of lipids present, this approach to quantitation is at best approximate with respect to accuracy.

Before examining quantitative comparisons of the different methods with respect to lipid class coverage, it is essential to note that although there are differences in both recovery and reproducibility of the 6 methods examined in this study, all methods showed recoveries >70% for all classes of lipids evaluated and run to run reproducibility <11% RSD. Thus, consistent, and precise results can be obtained in applications utilizing an individual sample preparation approach with a standard protocol and appropriate QC controls. 

Two specific important classes of lipids deserve further investigation. According to the interaction mechanism between EMR-Lipid sorbent and lipid molecules, certain lipid molecules lacking long linear acyl chains, such as cholesterol and short chain free fatty acids, may not be retained by the sorbent efficiently. Cholesterol was not characterized in this study and free fatty acids may have been poorly quantitated due to the lack of stable isotope labeled standards. Free fatty acids are difficult to measure quantitatively with LC-MS for a variety of reasons. They are often found as significant background contaminants originating from solvents and other sources, and the contaminants levels can be too high to mask the endogenous FFA in plasma. Another reason is that some free fatty acids could result from the degradation (hydrolysis) of complex lipids. However, collecting the flow-through from sample loading and washing steps can potentially partially recover these lipid classes. This may provide an alternative approach for analyses of these classes of lipids, but certainly needs further investigation.

#### 2.2.4. Comparisons Based on Methods Usability Characteristics

LLE sample preparation procedures are known to be time-consuming and labor-intensive. The common LLE methods for lipidomics sample preparation are even more tedious and troublesome due to small sample sizes, repeated extractions, phase separation, and challenging organic phase layer transfer, especially when the organic layer is at the bottom, as the case in the Bligh-Dyer and Folch protocols. When an emulsion occurs, phase separation and organic layer transfer could be even more difficult. For the Matyash LLE method, the organic layer stays on top, but caution must be taken not to aspirate any of the aqueous phase at the organic/aqueous interface. The single-phase BUME method precipitates the proteins and insoluble organic species. However, it compromises the cleanliness of the sample extract, as many polar to intermediate polar matrix interferences are co-extracted, similar as shown in Figure 2. This can result in ion suppression on some of the target lipid compounds.

By contrast, the Lipid Extraction SPE method simplifies the workflow, obviates the difficult phase separation and time-consuming transfer steps, and replaces multiple extractions with multiple elution steps. These features make the SPE procedure much easier and faster, more consistent, and user-friendly. In addition, the high selectivity and efficiency of the lipid retention mechanism on the SPE sorbent enable the use of a more efficient matrix clean-up, removing salts and other matrix co-extracted molecules without long aliphatic chains. 

There is a concern that aggressive solvents, such as dichloromethane and chloroform, could cause plastics leachable from SPE cartridges or plates. This is not an issue in traditional LLE methods, which typically utilize glass containers. The cleanliness of the cartridge/plate have been evaluated using reagent blank solvents and following the workflow. The results (see Supplementary Figure S3) demonstrated that minimal leachate was detected in the reagent blanks. Although there were minor peaks detected in the reagent blank obtained through the mock extraction procedure, none were identified as lipids in the current workflow. This is attributed to the cleanliness of the cartridge sorbent and materials and the short contact time for running solvents through the cartridge or plate. Furthermore, to minimize any potential leaching from collection devices, samples were always collected in glass tubes when SPE cartridge protocol was used, or glass-coated collection plates when SPE plate protocol was used. 

In terms of the time needed for preparation, the LLE methods required phase separation by centrifugation and manual transfers of organic layers that scale with the number of samples being extracted and multiple centrifugation steps. These steps added time and labor needed to conduct the procedure consistently. All methods required a drying step, which typically took between 30 min to 1 h. For the following time comparison for different methods, time needed for sample drying was not included. For a set of 48 samples, our experience was that the Bligh-Dyer, Folch, and Matyash methods require two to three hours of hands-on time. The single-phase BUME method is slightly faster, requiring one and half to two hours. This method obsoletes the difficult phase separation and transferring step but requires extensive centrifugation (60 min) to separate the supernatant and precipitates. By contrast, the Lipid Extraction SPE method can process 48 samples in 30–45 min. 

The SPE procedures are essentially additions of solvents to cartridges or wells of plates and do not require low expertise or specialized technique. This means that the methods can be executed by any operator and yield similar results. By contrast, LLE methodologies require a certain degree of expertise and results may vary depending on the individual’s level of expertise performing the extraction. 

Ultimately, the SPE plate procedure is well suited to automation. Further work is underway to optimize procedures based on SPE Lipid extraction for automation. The sequential recovery of both lipids and polar metabolites from individual mammalian cell samples using a combined protocol has recently been reported [32], which also demonstrated the applicability of the 96-well plate SPE workflow to an automated platform. 

## 3. Materials and Methods

### 3.1. Materials

Acetonitrile (ACN), methanol (MeOH), isopropanol (IPA), and chloroform were Chromosolv Brand LC-MS solvents obtained from Honeywell Research Chemicals (Muskegon, MI, USA). Butanol (BuOH), tert-butyl methyl ether (MTBE), and ammonium acetate were HPLC-Grade obtained from Sigma-Aldrich (St. Louis, MO, USA). HPLC grade water was produced using a MilliQ Water purification system from Millipore Sigma (Burlington, MA, USA). SRM 1950 Metabolites in Human Plasma NIST Standard was obtained from Sigma-Aldrich Chemical Company (St. Louis, MO, USA). UltimateSPLASH™ One and EquiSPLASH^®^ Internal Standards were obtained from Avanti Polar Lipids, Inc (Alabaster, AL, USA). WebSeal Plate+ 96-Well Glass-Coated Microplates (2.4 mL) were obtained from ThermoFischer (Waltham, MA, USA).

### 3.2. Deuterium Labeled Internal Standards Addition Procedures

For deuterium labeled internal standards recovery study, five samples were prepared as pre-spikes and five samples were prepared as post-spikes, using each extraction procedure. Preliminary experiments (not shown) have indicated that UltimateSPLASH™ One Internal Standard can be detected at the dilution level of 1:10 in plasma robustly and reliably (2.5–15 μg/mL depending on the lipid). To prepare a pre-spiked plasma sample at this level without affecting the solvent characteristics for the different sample preparations techniques, 100 μL aliquots UltimateSPLASH™ One Internal Standard was initially dried down, and reconstituted in 1000 μL SRM 1950 plasma. This mixture was vortexed and ultrasonicated for 10 min on ice and stored at −80 °C until use. To prepare the post-spike samples, 100 μL aliquot of UltimateSPLASH™ One Internal Standard was initially dried down and then reconstituted in 1 mL 1:1 BuOH/MeOH. This solution was vortexed and ultrasonicated for and was used to reconstitute dried post-spike plasma samples.

For the lipids profiling studies, all human plasma samples were extracted following the methods procedure, and then reconstituted in a solution containing EquiSPLASH^®^ Internal Standard in 1:1 BuOH/MeOH. To prepare the reconstituted solution, an aliquot of 100 μL EquiSPLASH^®^ Internal Standard was initially dried down and then reconstituted in 1 mL 1:1 BuOH/MeOH. This mixture was vortexed and ultrasonicated for 10 min on ice.

### 3.3. Lipid Extraction LLE Protocols

Two sets of lipid extractions were performed: one for the recovery study and one for the lipids profiling study. Except for the deuterium labeled internal standard addition procedure, the lipid extraction procedures were identical for samples used for both studies. The four different LLE methods were compared with the Lipid Extraction SPE approaches. The experimental design and workflow are summarized in Figure 3. For each method, 100 μL of SRM 1950 plasma was prepared in five replicates. For all methods, after extraction and drying steps, the dried samples were reconstituted into 100 µL 1:1 BuOH/MeOH and vortexed. The samples were then ready for LC-MS analysis or stored at −80 °C.

#### 3.3.1. Bligh-Dyer LLE Procedure [1]

Briefly, for each 100 μL plasma sample, an aliquot of ice-cold 1.4 mL chloroform/MeOH (1:1, *v*/*v*) was added directly to the sample in a glass tube. The suspension was vortexed and then incubated on ice for 30 min. With the addition of 0.6 mL water, the sample was vortexed and incubated on ice for an additional 10 min. The sample was centrifuged at 2000× *g* for 5 min at 4 °C. The bottom (organic) layer was transferred to a new glass tube. The aqueous layer was re-extracted with 1 mL of chloroform/MeOH (1:1, *v*/*v*), following the above procedure. The organic layers were combined and dried with N_2_ at 30 °C. 

#### 3.3.2. Folch LLE Procedure [1]

Briefly, for each 100 μL plasma sample, an aliquot of ice-cold 1.4 mL chloroform/MeOH (2:1, *v*/*v*) was added directly to the sample in a glass tube. The sample was vortexed and incubated on ice for 30 min. After the addition of 0.4 mL water, the sample was vortexed and incubated on ice for an additional 10 min. The sample was centrifuged at 2000× *g* for 5 min at 4 °C. The bottom (organic) layer was transferred to a new glass tube. The aqueous layer was re-extracted with 1 mL of chloroform/MeOH (2:1, *v*/*v*) following the above procedure. The organic layers were combined and dried with N_2_ at 30 °C.

#### 3.3.3. Matyash LLE Procedure [1]

Briefly, for each 100 μL plasma sample, an aliquot of ice-cold 4.2 mL MTBE/MeOH (2:1, *v*/*v*) was added directly to the sample in a glass tube. The sample was vortexed and incubated on ice for 30 min. After the addition of 1 mL water, the sample mixture was vortexed and incubated on ice for an additional 10 min. The sample was centrifuged at 2000× *g* for 5 min at 4 °C. The upper (organic) layer was transferred to a new tube. The aqueous layer was re-extracted with 1 mL of MTBE/MeOH/water (10:3:2.5:1 *v*/*v*) following the above procedure. The organic layers were combined and dried with N_2_ at 30 °C.

#### 3.3.4. BUME LLE Procedure [7]

Briefly, for each 100 μL plasma sample, an aliquot of 900 μL of ice-cold BuOH/MeOH (1:1, *v*/*v*) was added to the sample. The sample was vortexed and sonicated on ice for 60 min. The sample was centrifuged at 5000× *g* for 60 min. The supernatant was removed and dried with N_2_ at 30 °C.

### 3.4. Lipid Extraction SPE Protocol

#### 3.4.1. Lipid Extraction SPE on 1 mL Cartridges

An aliquot of 100 µL SRM 1950 plasma was transferred into a 2 mL polypropylene snap cap vial. An aliquot of 900 µL ice-cold ACN/MeOH (95:5, *v*/*v*) was added to the sample for protein precipitation. The tube was vortexed for 30 s and sonicated for 10 min on ice. Sonication assists with efficient protein precipitation, as well as the release of any trapped lipids from protein precipitates. The Bond Elut Lipid Extraction 1 mL cartridges (Agilent Technologies, Wilmington, DE, USA), containing 40 mg sorbent per cartridge were placed on a Positive Pressure Manifold 48 Processor (PPM-48, Agilent Technologies, Wilmington, DE, USA) with a waste reservoir under the cartridges. Each sample was vortexed again for 10 s, and the entire homogenate was transferred onto the SPE 1 mL cartridge using a wide-bore pipette tip.

Slow elution with a flow rate of 3–5 s/drop was generated by gravity. The steady elution flow rate allows for sufficient interaction time between lipid compounds and the sorbent, so that lipids can be retained on the sorbent efficiently. After all visible liquid had eluted, an aliquot of 1 mL of ACN/H_2_O (9:1, *v*/*v*) was added for sample washing by gravity or with low pressure (1–3 psi) when needed. The above washing step was repeated one more time. When no visible liquid was left in the cartridge, higher pressure (15 psi) was applied to dry the sorbent bed. The waste reservoir was removed, and glass collection tubes were placed under cartridges. An aliquot of 1 mL DCM/MeOH (1:2 *v*/*v*) was added to each cartridge for gravity elution or with low pressure, 1–3 psi, as needed. A second aliquot of 1 mL DCM/MeOH (1:2 *v*/*v*) was added for additional gravity elution. High pressure (6–9 psi) was applied at the end to dry the sorbent completely. The entire eluent was dried with N_2_ at 30 °C.

#### 3.4.2. Lipid Extraction SPE Protocol on 96 Well Plates

For the purposes of this study, 1000 µL SRM 1950 plasma was transferred into a 15 mL glass test tube. An aliquot of 9000 µL ice-cold ACN/MeOH (95:5, *v*/*v*) was added to the sample for protein precipitation. The tube was vortexed for 30 s and sonicated for 10 min on ice. Sonication assists with efficient protein precipitation, as well as the release of any trapped lipids from protein precipitates. This step can be conducted in a 2 mL collection plate, if the multi-probe pipette or robotic 96-probe pipette is available in lab. The Bond Elut Lipid Extraction 96 well plates (Agilent Technologies, Wilmington, DE, USA), containing 60 mg sorbent per well, were placed on a Positive Pressure Manifold 96 Processor (PPM-96, Agilent Technologies, Wilmington, DE, USA) with a waste reservoir under the plate. Each sample was vortexed again for 10 s, and the entire homogenates was transferred into individual wells of the 96 well plates using a wide-bore pipette tip. 

Slow elution with a flow rate of 3–5 s/drop was generated by gravity. An aliquot of 1 mL of ACN/water (9:1, *v*/*v*) was added for sample washing using gravity or low pressure (1–3 psi) as needed. The above washing step was repeated one more time. When no visible liquid was left in the cartridge, higher pressure (15 psi) was applied to dry the cartridge. The waste reservoir was removed and replaced with a 2.4 mL glass coated deep well collection plate. An aliquot of 1 mL DCM/MeOH (1:2 *v*/*v*) was added to each well for gravity elution or with low pressure, 1–3 psi, as needed. A second aliquot of DCM/MeOH (1:2 *v*/*v*) was added for additional gravity elution. High pressure (6–9 psi) was applied at the end to dry the sorbent bed completely. The entire eluent was dried with N_2_ at 30 °C.

### 3.5. Lipid Analysis by LC-MS/MS

LC-MS/MS was conducted using an Agilent 6490 LC/TQ equipped with a Jet Stream Technology ionization source and operated with the settings listed in Supplementary Table S1. Lipid chromatographic separations were conducted on an Agilent 1290 Infinity II UHPLC system consisting of a 1290 Infinity II HiSpeed (binary) Pump, 1290 Infinity II Multisampler, and 1290 Infinity II Multicolumn Thermostat.

The MS/MS acquisition approach was based on a “dynamic Multiple Reaction Monitoring” (dMRM) method which targeted 63 specific deuterium labeled lipids. The dMRM conditions are listed in Supplementary Table S2. Transition conditions and production ion structures were determined with the aid of LipidCreator open-source software [33]. Five preparation replicate samples were made for each preparation method and 5 LC-MS replicate injections were analyzed for each sample. The LC-MS/MS analysis order was randomized with respect to sample. 

For rapid evaluation and comparison of different parameter settings’ impact on the lipid extraction efficiency, the total phosphatidylcholine/sphingomyelin profile was monitored as a surrogate for polar lipid profiles using precursor ion scan of the *m*/*z* 184.1 fragment (protonated phosphocholine) with scan range *m*/*z* 100–1300.

The lipid concentrations were calculated using MassHunter Quantitative Analysis Ver 10.2 against a seven-point calibration for the range of 0.01 to 1× dilution of UltimateSPLASH™ One Internal Standard Mixture in a Folch style extraction of SRM 1950 plasma. Because the individual lipids are present in different levels in the mixture, this spans a concentration level of 0.25–150 μg/mL depending on the lipid. 

### 3.6. Lipid Profiling by LC-HRMS and LC-HRMS/MS

LC-HRMS and LC-HRMS/MS were conducted using an Agilent 6545 LC/QTOF equipped with a Jet Stream Technology ionization source operated under the settings listed in Supplementary Table S3. Lipid chromatographic separations were conducted with the same LC system as described in Section 3.5.

The analytical strategy consisted of a two-stage process outlined in Figure 3 and described in depth in other reference [34]. In the first stage, a pooled sample consisting of an equivalent aliquot from each of the six preparation methods was analyzed by LC-HRMS/MS. A series of Iterative LC-HRMS/MS analyses were made on the pooled sample in both positive and negative mode and used to generate a complete, accurate mass and retention time database of lipids detected in any of the samples. In the second stage, samples prepared by each preparation method were profiled in triplicate using LC-HRMS in both positive and negative modes to annotate lipids against the accurate mass and retention time database in a targeted data analysis process. 

Iterative HRMS/MS data acquisition was made on the pooled sample to generate an accurate mass and retention time database against which to profile the lipids in the individual samples. Auto HRMS/MS in both positive and negative modes was conducted using the method conditions listed in Table 1. In Iterative HRMS/MS, Auto HRMS/MS conditions during each acquisition follow standard data-dependent acquisition parameters, including active exclusion of precursors to maximize the number of unique HRMS/MS spectra recorded within each acquisition. However, in Iterative HRMS/MS, after the first acquisition was complete and for each subsequent acquisition, precursors already selected for HRMS/MS were added to a rolling exclusion list and were excluded from further and redundant isolation, allowing for more efficient collection of unique HRMS/MS spectra for a given sample. Functionally, Iterative HRMS/MS extends active exclusion across a series of multiple injections. Valid precursors of low abundance that might otherwise be missed are often successfully acquired with Iterative HRMS/MS. Details of the Iterative HRMS/MS protocol are illustrated in Supplementary Figure S4.

### 3.7. Data Processing

LC-HRMS/MS data from the pooled sample were annotated to specific lipids using HRMS/MS in silico spectral matching with the MassHunter Lipid Annotator 1.0 software (Agilent Technologies). This software uses an algorithm that combines probability density, Bayesian scoring, and a non-negative least square fit to search a theoretical lipid library [35]. Positive and negative mode datasets were treated separately throughout data analysis.

Individual features in the LC-HRMS data for each of sample preparation methods were extracted and aligned using MassHunter Profinder version 10.0 (Agilent Technologies), searching in a batch targeted feature extraction (BTFE) mode against the PCDL (personal compound database library) generated from the pooled sample in Lipid Annotator, requiring both mass and retention time criteria. The resulting features were exported to Mass Profiler Professional (MPP) Version 15.0 (Agilent Technologies) for lipid class and statistical analysis. The abundance data were normalized for lipid class using the EquiSPLASH^®^ Internal Standard Mixture. Specific lipids were normalized using the deuterium labeled internal standards from same lipid class. If no class standard was present, an average normalization of all standards was used. Individual features were filtered according to the following criteria; (a) features were included if at least 100.0% of samples in any 1 out of 6 conditions have flags are “present”(b) features where at least 1 out of 6 conditions have CV < 25.0% (c) features with CV < 25.0%. Additional plotting and statistical analysis was done with OriginPro, Version 2021 (OriginLab Corporation, Northampton, MA, USA).

The lipid concentrations were calculated using MassHunter Quantitative Analysis against a seven-point calibration range of 0.01 to 1× dilution of EquiSPLASH^®^ Internal Standard Mixture in a Folch style extraction of SRM 1950 human plasma. This spans a concentration range of 1–100 μg/mL for each lipid. Specific lipids were calibrated using the deuterium labeled internal standards from the same lipid class. If no class standard was present, an average calibration of all standards was used.

## 4. Conclusions

A novel sample preparation SPE method using Lipid Extraction SPE in both cartridge and 96 well plate format was developed and evaluated for human plasma lipid profiling by LC-MS. The Lipid Extraction SPE method demonstrated comparable qualitative and quantitative results to the four conventional LLE methods (Bligh-Dyer, Folch, Matyash, and BUME) in terms of lipids recovery and reproducibility, and the lipid class distribution. The new method provides advantages in terms of ease of use, time and labor-saving, and improved reproducibility. 

## Figures and Tables

**Table 1:** Optimization of SPE elution solvents based on *m*/*z* 184 precursor-ion scans.

Final Elution Investigation (n = 3 or 4)	Total PPLs Recovery (RSD%)
2:1 Chloroform/MeOH, 2 × 1 mL	107 (4.1%)
1:1 Chloroform/MeOH, 2 × 1 mL	112 (0.6%)
1:2 Chloroform/MeOH, 2 × 1mL	92 (2.0%)
2:1 MTBE/MeOH, 2 × 1 mL	8 (86.8%)
1:1 MTBE/MeOH, 2 × 1 mL	31 (15.4%)
1:2 MTBE/MeOH, 2 × 1 mL	46 (3.1%)
2:1 DCM/MeOH, 2 × 1 mL	84 (6.1%)
1:1 DCM/MeOH, 2 × 1 mL	102 (12.3%)
1:2 DCM/MeOH, 2 × 1 mL	116 (0.4%)
2:1 1-Chlorobutane/MeOH, 2 × 1 mL	103 (8.1%)
1:1 1-Chlorobutane/MeOH, 2 × 1 mL	104 (13.1%)
1:2 1-Chlorobutane/MeOH, 2 × 1 mL	95 (2.1%)

**Table 2:** Concentration and count of identified lipids.

		SPE Cartridge		SPE Plate		LLE-Bligh-Dyer		LLE-Folch		LLE-Matyash		LLE-BUME	
Mode	Class	Summed Conc	#Lipids	Summed Conc	#Lipids	Summed Conc	#Lipids	Summed Conc	#Lipids	Summed Conc	#Lipids	Summed Conc	#Lipids
		(nmol/mL)		(nmol/mL)		(nmol/mL)		(nmol/mL)		(nmol/mL)		(nmol/mL)	
Positive	ACar	1.12	9	1.61	8	1.01	8	0.94	10	0.91	9	0.35	6
	CE	1.32	9	1.75	9	1.01	7	1.10	8	0.66	7	0.26	8
	Cer_NS	3.34 × 10^−5^	5	1.50 × 10^−4^	7	1.34 × 10^−5^	4	3.12 × 10^−5^	6	8.18 × 10^−5^	5	2.38 × 10^−5^	3
	DG	4.35 × 10^−2^	8	4.54 × 10^−2^	8	1.92 × 10^−2^	6	2.44 × 10^−2^	8	1.27 × 10^−2^	6	5.13 × 10^−2^	8
	HexCer_NS	5.05 × 10^−6^	2	1.86 × 10^−5^	2	2.99 × 10^−5^	1	3.22 × 10^−5^	2	2.67 × 10^−5^	3	2.48 × 10^−5^	3
	LPC	0.26	61	0.19	57	0.40	50	0.33	60	0.35	58	0.24	57
	PC	2.73	75	4.93	80	5.16	66	5.40 × 10^3^	80	4.81	80	1.85	61
	SM	0.41	38	0.39	40	0.55	32	0.63	40	0.43	42	0.26	37
	TG	1.27	31	1.53	31	0.73	26	1.15	31	1.05	28	0.51	29
Negative	Cer_NS	2.20 × 10^−2^	22	7.56 × 10^−2^	25	0.11	23	0.11	25	0.15	22	7.52 × 10^−2^	24
	EtherPC	9.43 × 10^−3^	13	6.76 × 10^−3^	10	1.23 × 10^−2^	11	1.27 × 10^−2^	10	1.35 × 10^−2^	9	7.96 × 10^−3^	9
	EtherPE	3.17 × 10^−2^	4	4.51 × 10^−2^	3	7.31 × 10^−2^	5	7.69 × 10^−2^	5	5.09 × 10^−2^	4	7.96 × 10^−3^	4
	FA	6.57 × 10^−2^	3	7.88 × 10^−2^	4	0.19	4	0.11	4	0.14	4	0.13	4
	HexCer_NS	9.27 × 10^−3^	2	9.58 × 10^−3^	2	2.31 × 10^−2^	2	2.59 × 10^−2^	2	1.15 × 10^−2^	2	2.14 × 10^−2^	2
	LPC	1.57 × 10^−2^	13	6.75 × 10^−3^	11	1.51 × 10^−2^	13	1.33 × 10^−2^	13	1.12 × 10^−2^	13	9.81 × 10^−3^	13
	LPE	9.27 × 10^−3^	2	3.34 × 10^−3^	4	3.47 × 10^−3^	4	3.67× 10^−2^	4	1.86 × 10^−2^	5	2.65 × 10^−2^	5
	LPI	9.32 × 10^−5^	2	2.66 × 10^−5^	1	5.16 × 10^−5^	1	5.15 × 10^−3^	1	5.34 × 10^−5^	1	4.51 × 10^−4^	2
	OxPC	1.41 × 10^−2^	3	1.94 × 10^−2^	3	3.38 × 10^−2^	2	3.54 × 10^−2^	3	2.20 × 10^−2^	3	1.07 × 10^−2^	2
	PC	0.42	33	0.62	34	1.10	32	1.16	33	0.79	30	0.34	31
	PE	3.44 × 10^−2^	8	3.68 × 10^−2^	9	7.68 × 10^−2^	8	8.60 × 10^−2^	7	4.52 × 10^−2^	9	4.42 × 10^−2^	8
	PI	1.19 × 10^−2^	11	2.04 × 10^−2^	10	4.69 × 10^−2^	11	4.73 × 10^−2^	12	6.31 × 10^−2^	11	3.65 × 10^−2^	9
	PS	5.51 × 10^−2^	6	8.07 × 10^−2^	6	0.14	6	0.15	6	0.10	6	4.75 × 10^−2^	6
	SM	5.12 × 10^−2^	17	6.01 × 10^−2^	17	0.23	19	0.27	19	0.30	17	0.17	18
	Total		377		381		341		389		374		331

## Data Availability

The data presented in this study are available on request from the corresponding author.

## Supplementary Materials

The following are available online at https://www.mdpi.com/article/10.3390/metabo11050294/s1, Table S1: LC-MS/MS Method Conditions on LC/TQ, Table S2: Deuterium labeled lipids internal standards dMRM parameters (UltimateSPLASH™ One), Table S3: LC-HRMS and LC-HRMS/MS Method Conditions on LC/QTOF, Figure S1a,b: Recovery data for the individual lipids, Figure S2: Negative Ion Clustering by LC-HRMS/MS method, Figure S3: Comparison of plasma and mock extractions with Bond Elut Lipid Extraction Plates, Figure S4: Iterative MSMS for pooled plasma samples.

## Acronyms

Acar	acylcarnitine
ACN	acetonitrile
BUME	butanol-methanol
BuOH	butanol
CE	cholesterol ester
Cer	ceramide
Cer_NS	ceramide containing nonhydroxy-fatty acid-sphingosine
DCM	dichloromethane
DG	diacylglyceride
EMR	Enhance Matrix Removal
EtherPC	ether phosphatidylcholine
EtherPE	etherphosphatidylethanoloamine
FA	fatty acid
HexCer_NS	hexosyl-ceramide
IPA	Isopropanol
LC	liquid chromatography
LC/TQ	liquid chromatography-Tandem Quadrupole Mass Spectrometry
LC-HRMS	liquid chromatography-high resolution mass spectrometry
LC-HRMS/MS	liquid chromatography-high resolution mass spectrometry/mass spectrometry
LC-MS	Generic liquid chromatography-mass spectrometry
LLE	liquid liquid extraction
LPC	lysophosphatidylcholine
LPE	lysophosphatidylethanolamine
LPI	lysophosphatidylinisitol
LPL	lysophospholipid
MeOH	Methanol
MG	monoglyceride
MPP	mass profiler professional
MTBE	tert-butyl methyl ether
NIST	National Institute of Standards and Technology
OxPC	oxidized phosphatidylcholine
PA	phosphatidic acid
PC	phosphatidylcholine
PCDL	personal Compound Database Library
PE	phosphatidylethanolamne
PG	phosphatidylglycerol
PI	phosphatidylinositol
ppt	precipitate
PS	phosphatidylserine
QTOF	quadrupole time of flight
RT	retention time
SM	sphingomyelin
SPE	solid phase extraction
SRM	standard refernce material
TG	triglyceride
TIC	total ion chromatogram
