# Supplementary material for: A Novel Solid Phase Extraction Sample Preparation Method for Lipidomic Analysis of Human Plasma Using Liquid Chromatography/Mass Spectrometry
Source: Metabolites. 2021 May 4;11(5):294. doi: 10.3390/metabo11050294 (PMC8147762; doi:10.3390/metabo11050294)
Supplement: Supplementary file 1 [file metabolites-11-00294-s001.zip › Apffel et al-supplementary materials(Revised).pdf]

Supplementary Materials

Supplementary Table S1: Analytical Conditions for LC/TQ.

Parameter	Agilent 6490 LC/TQ with Dual Agilent Jet Stream Source	
**Instrument mode**	2 GHz, extended dynamic range, m/z 1,700	
**Polarity**	Positive and Negative	
**Gas temperature**	210 ºC	
**Drying gas (nitrogen)**	13 L/min	
**Nebulizer gas**	45 psi	
**Sheath gas**	250 °C @ 12 L/min	
**Capillary voltage**	3,500 V (+), 3,000 V (-)	
**Nozzle voltage**	500 V	
**Fragmentor**	160 V	
**Oct 1 Rf Vpp**	750 V	
**Acquisition Conditions**	See Supplementary Table S1b	
	**Agilent 1290 Infinity II LC**	
**Analytical column**	Agilent InfinityLab Poroshell EC-C18 2.0x100 mm, 1.9 µm (p/n 695675-902)	
**Column temperature**	60 ºC	
**Injection volume**	1 µL	
**Autosampler temperature**	5 ºC	
**Needle wash**	15 seconds in wash port (50:50 MeOH/IPA)	
**Mobile phase**	A) 10mM Ammonium Acetate, 10 uM Medronic Acid in 9:1 H_2O/MeOH<br>B) 10mM Ammonium Acetate in 3:2:5 ACN/MeOH/IPA	
**Flow rate**	0.4 mL/min	
**Gradient program**	Time (min)	%B
	0	62
	2.0	84
	8.0	86
	8.1	93
	10.0	100
	12.9	100
	13	62
**Stop time**	20 minutes	
**Post time**	0 minutes	

Supplementary Table S3: Analytical Conditions for LC/QTOF

Parameter	Agilent 6545 LC/QTOF with Dual Agilent Jet Stream Source
**Instrument mode**	2 GHz, extended dynamic range, m/z 1,700
**Polarity**	Positive and Negative
**Gas temperature**	210 ºC
**Drying gas (nitrogen)**	13 L/min
**Nebulizer gas**	45 psi
**Sheath gas**	250 °C @ 12 L/min
**Capillary voltage**	3,500 V (+), 3,000 V (-)
**Nozzle voltage**	0 V
**Fragmentor**	160 V
**Oct 1 Rf Vpp**	750 V
**Acquisition speed**	MS-Only: 3 spectra/second (MS)<br>Auto MS/MS: 3 spectra/second (MS), 4 spectra/second (MS/MS)
**Auto MS/MS parameters**	Isolation width: Narrow (~1.3 amu)<br>Collision energy: 20, 35 eV
**Reference correction**	2 points at m/z 121.050873(+), 922.009798(+)<br>2 points at m/z 119.036320(-), 980.016375(-)
	Agilent 1290 Infinity II LC
**Analytical column**	Agilent InfinityLab Poroshell EC-C18 2.0x100 mm, 1.9 µm (695675-902)
**Column temperature**	60 ºC
**Injection volume**	1 µL
**Autosampler temperature**	5 ºC
**Needle wash**	15 seconds in wash port (50:50 MeOH/IPA)
**Mobile phase**	A) 10 mM Ammonium Acetate, 10 µM Medronic Acid 9:1 H_2O/MeOH<br>B) 10 mM Ammonium Acetate, 3:2:5 ACN/MeOH/IPA
**Flow rate**	0.3 mL/min
**Gradient program**	Time(min) %B<br>0 60<br>0.5 60<br>3.0 57<br>9.5 87<br>10.5 95<br>14.0 96<br>14.5 100<br>20.0 100<br>20.1 60
**Stop time**	22 minutes
**Post time**	5 minutes

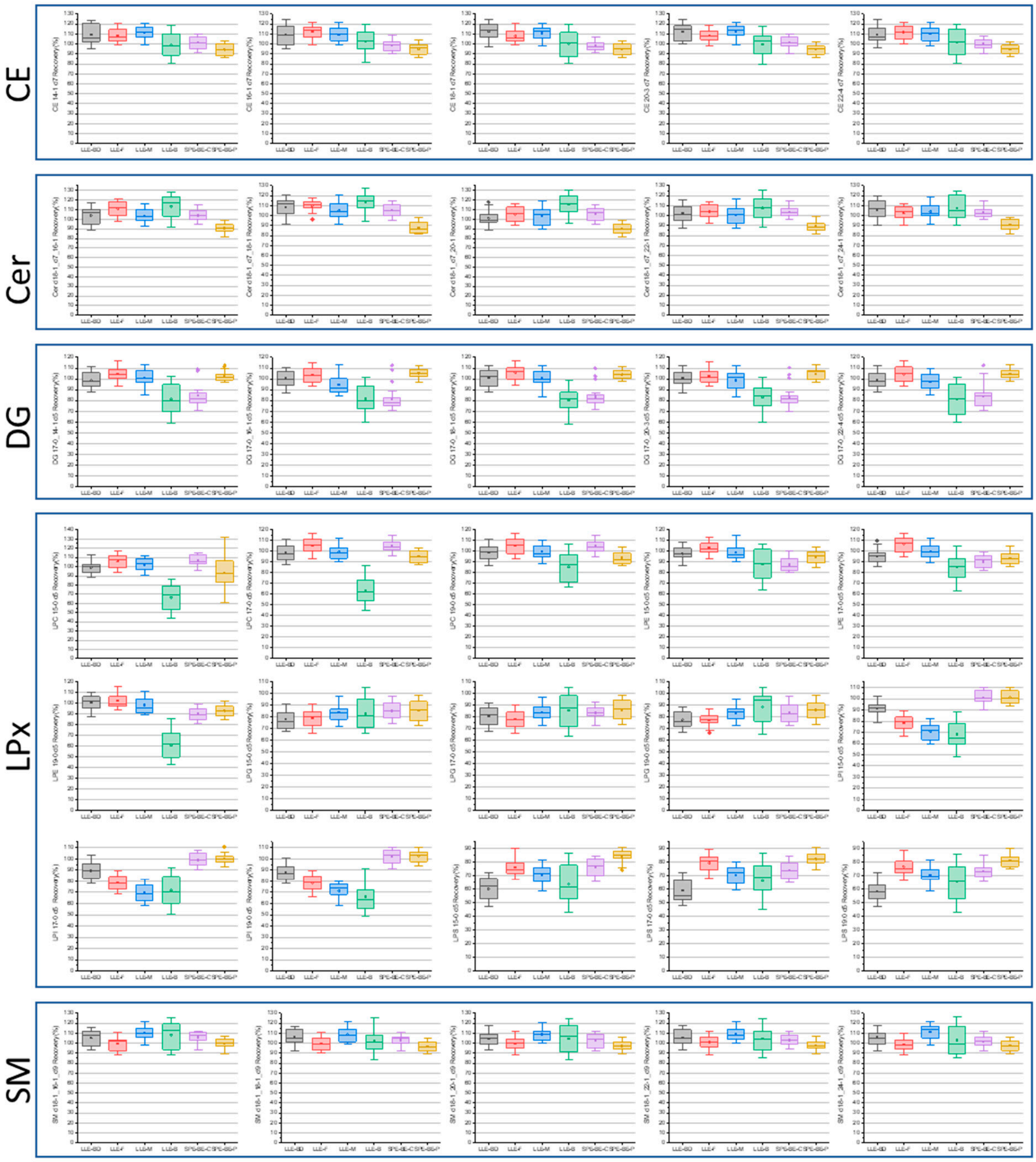

Supplementary Figure S1a: Recovery of stable labeled lipids internal standards based for individual lipids classes. Each bar represents the average of 5 preparation replicates x 5 MS replicates. recovery of stable labeled lipids internal standards based on lipids classes based on targeted LC-TQ workflow. In the box plots, the box represents the 1st to 3rd quartile to data with the central line denoting the median. The small square represents the mean and the whiskers are set at 5th and 95th percentile. Cholesterol esters(CE), ceramides(Cer), diacylglycerides (DG), lysophosphocholine(LPC), lysophosphoethanola-mine(LPE), lysophosphoglycerol(LPG), lysophosphoinosotol(LPI), lysophosphoserine(LPS), sphingolmyelin(SM).

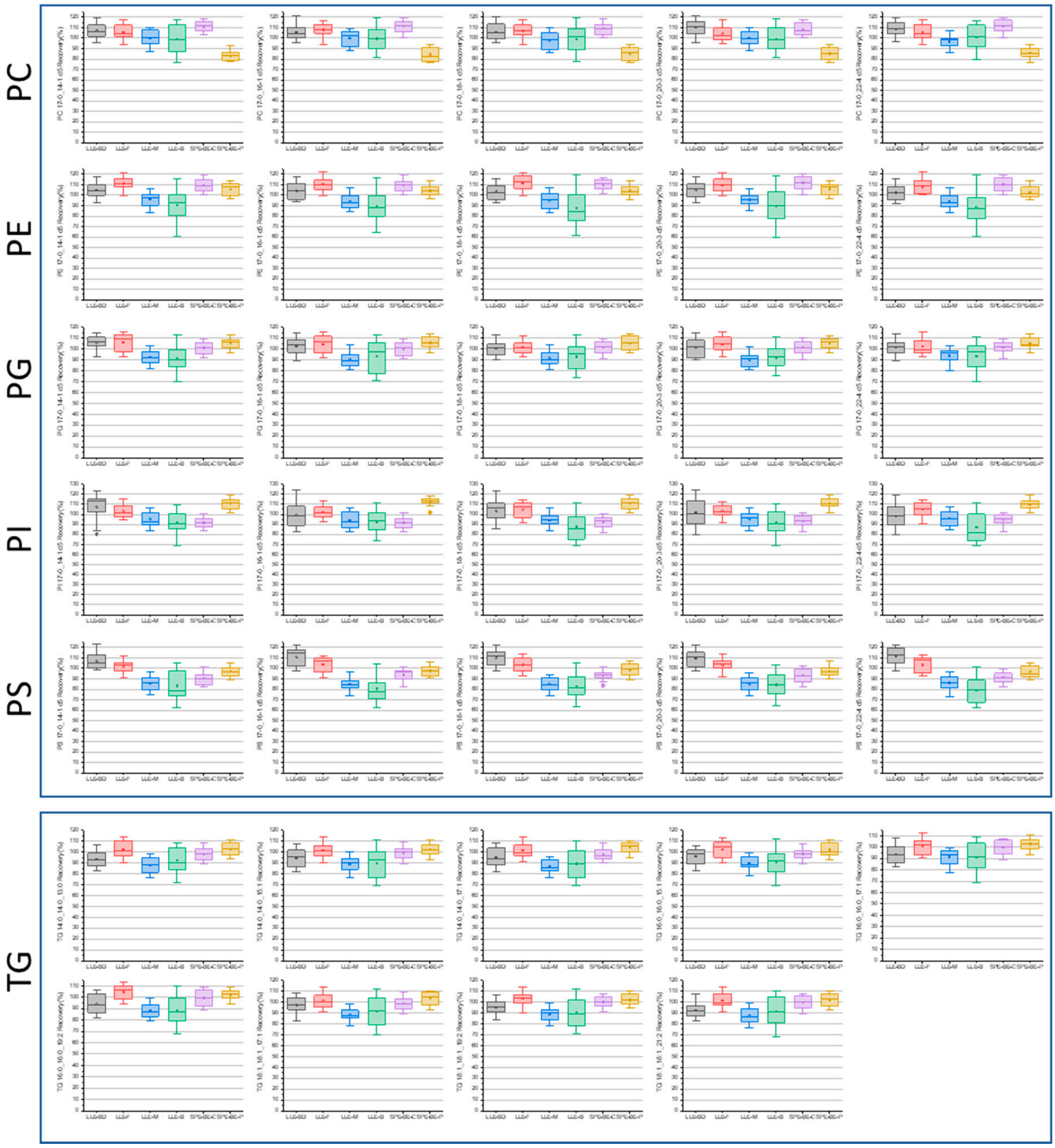

Supplementary Figure S1b: Recovery of stable labeled lipids internal standards based for individual lipids classes. Each bar represents the average of 5 preparation replicates x 5 MS replicates. recovery of stable labeled lipids internal standards based on lipids classes based on targeted LC-TQ workflow. In the box plots, the box represents the 1st to 3rd quartile to data with the central line denoting the median. The small square represents the mean and the whiskers are set at 5th and 95th percentile. Phosphatidylcholine(PC), phosphatidylethanolamine(PE), phosphatidylgycerol(PG), phosphatidylinosi-tol(PI), phosphatidylserine(PS), triacylglycerol(TG).

Supplementary Figure S2: Unsupervised hierarchical lipid clustering by different sample preparation

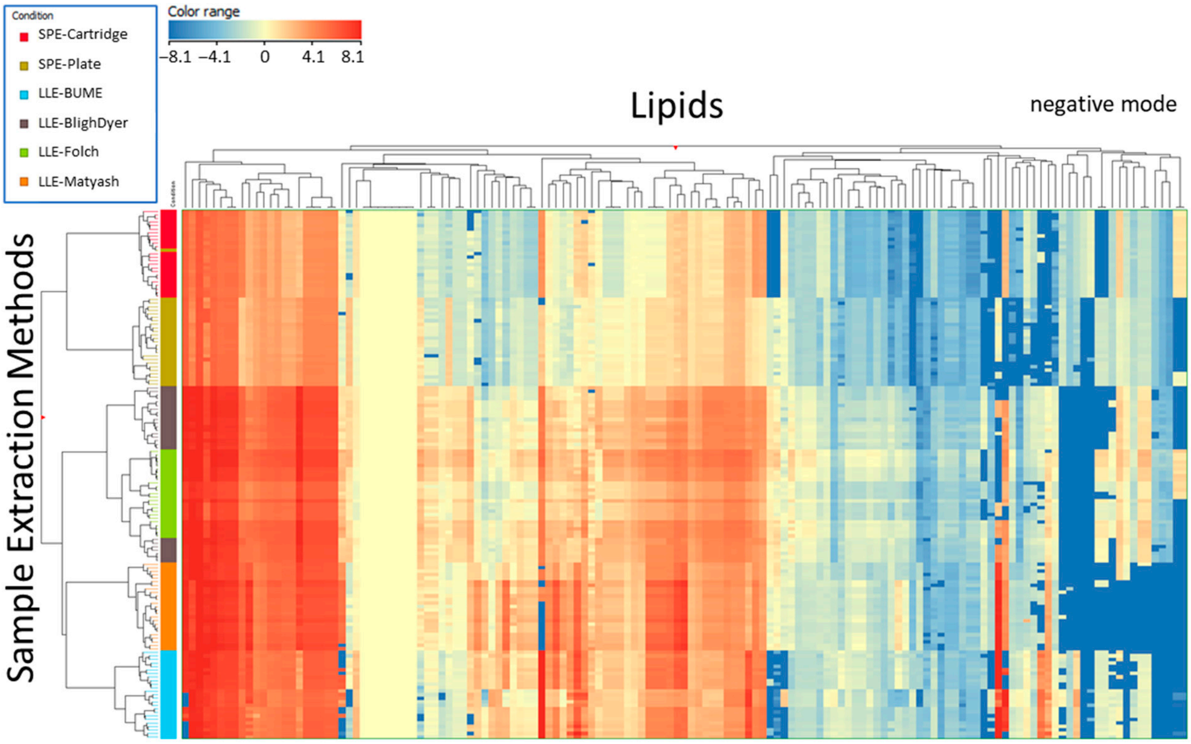

methods for negative ion mode LC-HRMS data. Each extraction method is represented by 5 preparation replicates x 5 analytical replicates. Log2 transformed abundance were normalized with respect to deuterium labeled lipid class standards (EquiSplash™).

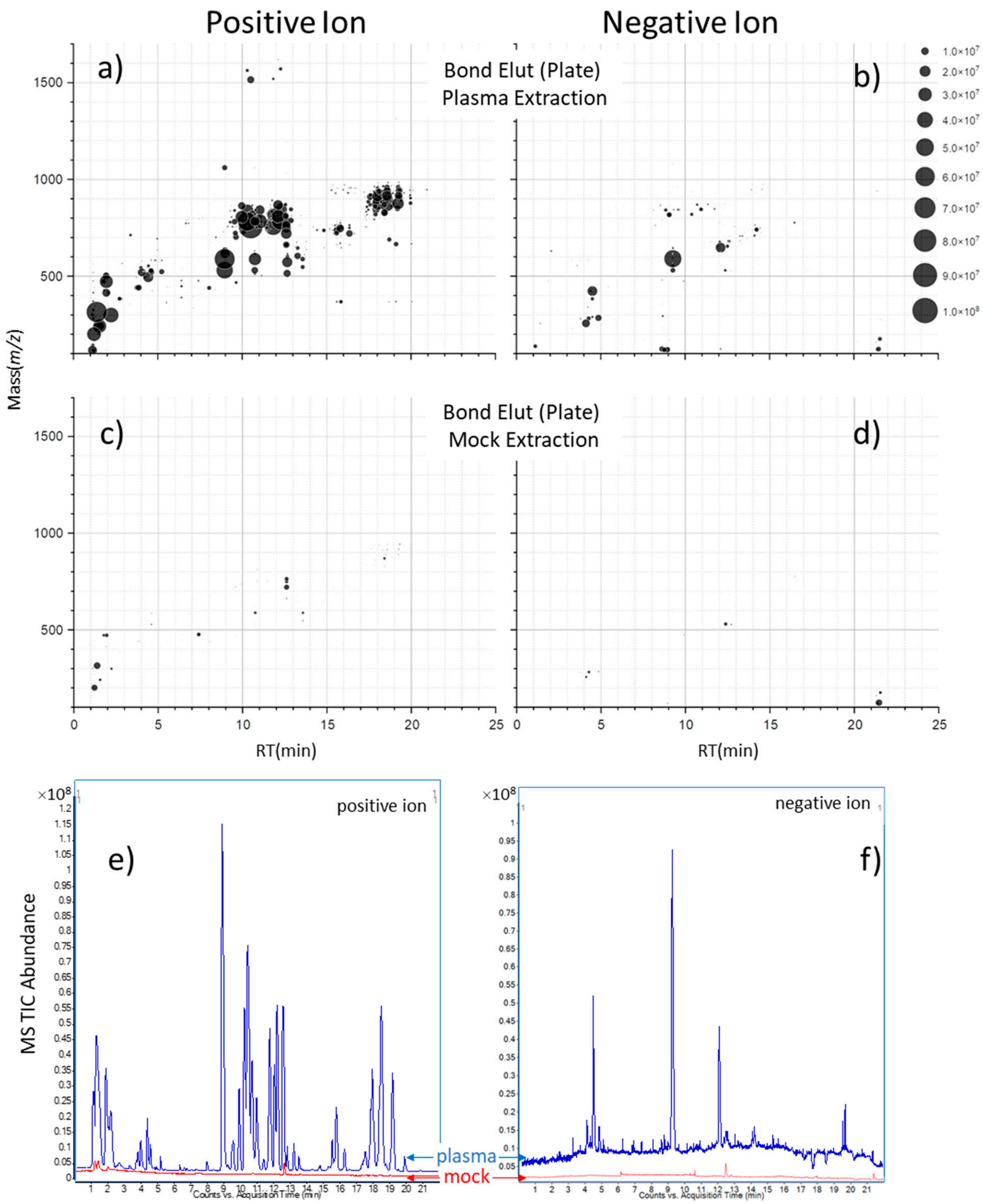

Supplementary Figure S3. Comparison of plasma and mock extractions with Bond Elut Lipid Extraction Plates. RT(min) vs Mass(m/z) for plasma extract in positive (a) and negative modes(b). RT(min) vs Mass(m/z) for mock extract in positive (c) and negative modes(d). Bubble size in a-d represent peak area as indicated in legend. Corresponding MS Total Ion Chromatograms are shown in positive ion (e) and (f), with TIC chromatograms from plasma sample prepared by SPE (red) and those from reagent blank prepared through muck SPE procedure (blue).

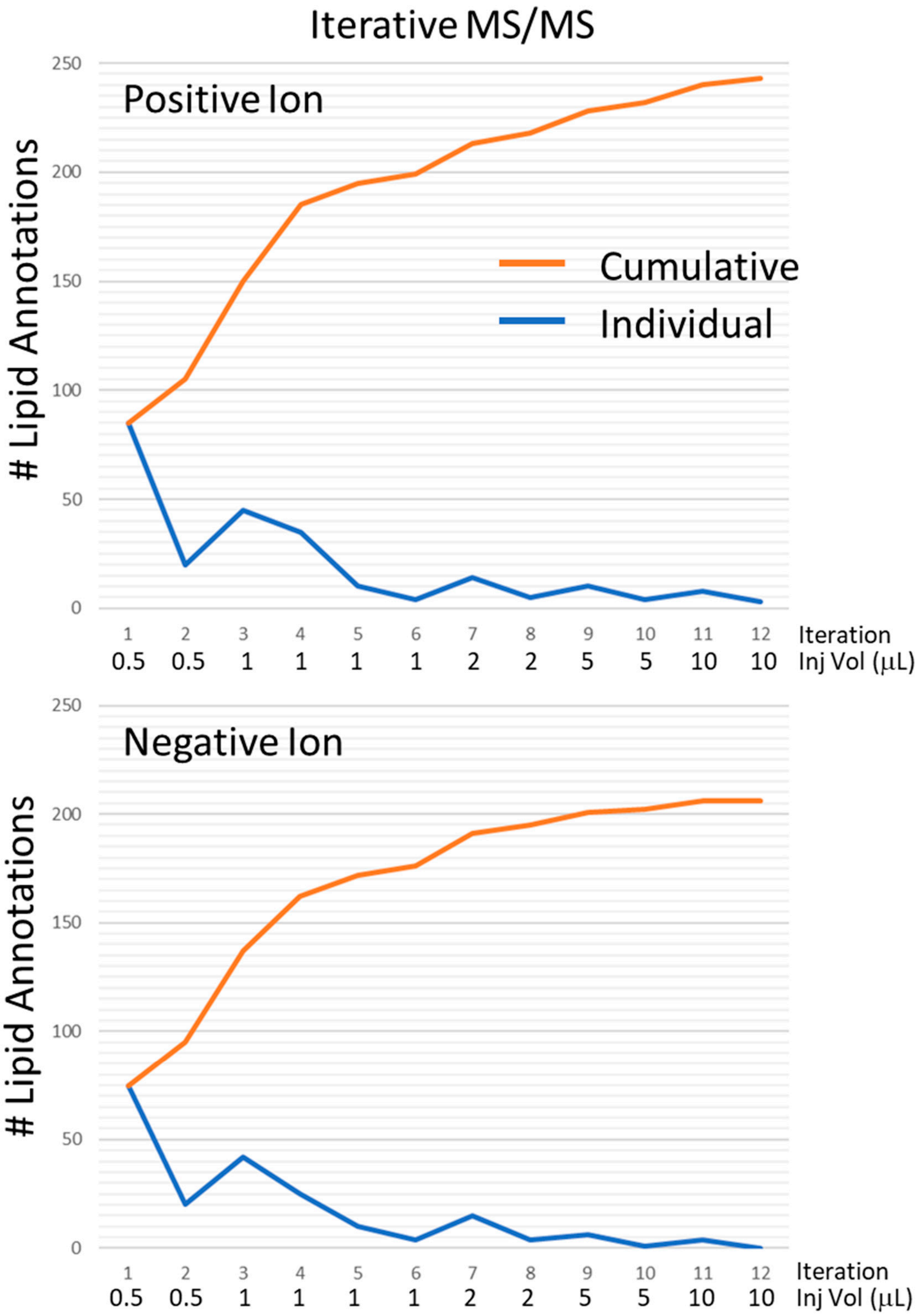

Supplementary Figure S4 . Iterative MSMS for pooled plasma samples. In the course of this study, the effect of # of iterations vs injection volume was evaluated. It was found that 12 iterations was more than necessary. As the # of iterations increase, the cumulative # annotated lipids (red line) increases. Considering the number of lipid annotations for each individual data file (blue line), at each point that the injection volume is increased, the # of new annotated lipids increases, but then drops.
